# Pheochromocytoma Crisis in the Emergency Department

**DOI:** 10.7759/cureus.13683

**Published:** 2021-03-03

**Authors:** Stephanie R Bartikoski, Daniel J Reschke

**Affiliations:** 1 Emergency Medicine, San Antonio Military Medical Center, San Antonio, USA

**Keywords:** pheochromocytoma, pheochromocytoma crisis, hypertensive emergency, pulmonary edema, pulmonary embolism, adrenal tumor, phentolamine

## Abstract

Pheochromocytoma is a rare, often undiagnosed adrenal tumor that typically presents in early adulthood and is characterized by intermittent surges of catecholamines. While this “Great Mimic” may present with a variety of vague complaints such as headache, abdominal pain, or palpitations, it may also appear as a severely hypertensive patient with multi-organ failure and cardiopulmonary collapse known as pheochromocytoma crisis. Management of hypertensive emergency in these patients is unique, and the associated metabolic derangements, coagulopathy, thromboembolic events, and risk of adrenal capsule rupture add significant complexity, morbidity, and mortality to these cases. Emergency providers should learn when to suspect this uncommon but life-threatening diagnosis in order to properly manage these potentially critically ill patients.

## Introduction

Pheochromocytomas are chromaffin cell tumors originating from neural crest cells [1]. They may occur spontaneously or have a hereditary component with an annual incidence of 2 to 9.1 per 1 million adults typically between the third and fifth decades of life with almost half being diagnosed at autopsy [1]. While most pheochromocytomas are benign, about one-quarter are malignant [1].

The tumor releases intermittent surges of catecholamines which can present clinically with vague complaints such as headache, palpitations, fatigue, nausea, tremulousness, weight loss, or abdominal pain. Occasionally, patients may present with hemodynamic instability and end-organ dysfunction known as pheochromocytoma crisis. While the crisis may occur as part of the natural disease process, it can also be precipitated suddenly by general anesthesia, direct trauma to the tumor, or with the administration of metoclopramide [2].

## Case presentation

A healthy 30-year-old male presented to the emergency department (ED) after waking with sudden epigastric pain, nausea, and multiple episodes of vomiting. He also noted palpitations, shortness of breath, and orthopnea on arrival. He denied any thyroid problems or stimulant drug use. Vital signs showed a pulse of 139 beats per minute, blood pressure of 208/161 mm Hg, a respiratory rate of 30 breaths per minute with an oxygen saturation of 86% on room air, and his temperature was 98.8 degrees Fahrenheit. On exam, the patient was in obvious respiratory distress and placed on supplemental oxygen. Bedside ultrasound revealed diffuse B-lines and a hyperdynamic myocardium. Electrocardiogram (EKG) showed sinus tachycardia.

Treatment for hypertensive emergency with flash pulmonary edema was initiated with intravenous nicardipine at 5 mg per hour and intravenous nitroglycerin at 20 mcg per minute with titration to 50 mcg per minute. He was placed on noninvasive ventilatory support promptly once available with an inspiratory pressure of 10 cm of water and an expiratory pressure of 5 cm of water. Despite these efforts, he became agitated, noncompliant with the face mask, increasingly tachycardic, and developed severe angina with worsening mixed respiratory failure. The patient was subsequently intubated with copious pink, frothy secretions noted.

**Figure 1:**
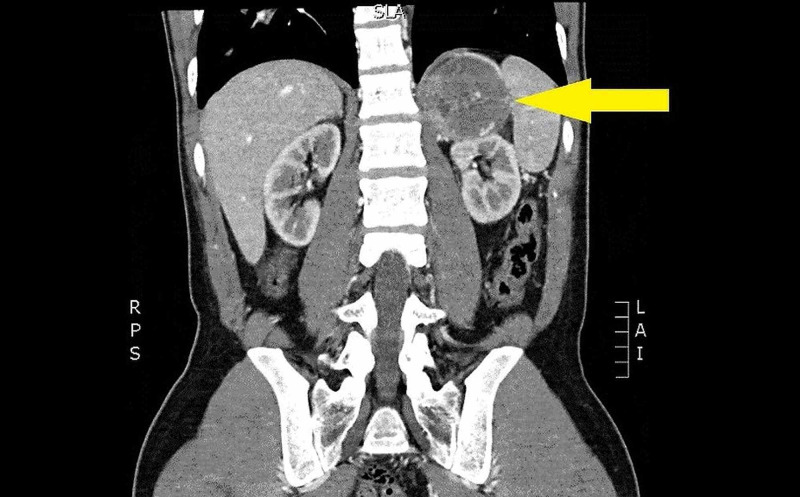
Left adrenal mass measuring 6.5 cm

CT imaging showed a 6.5 cm left adrenal mass (Figure 1) and multiple bilateral segmental pulmonary emboli with pulmonary infarct. Laboratory investigations were notable for marked hyperglycemia of 489 mg/dL, acidosis with a pH of 7.2 with an anion gap of 22, thrombocytopenia to 257/microliter, fibrinogen 60mg/dL, prothrombin time (PT) of 24.4 seconds, partial thromboplastin time (PTT) of 104 seconds, international normalized ratio (INR) 2.2, d-dimer >20 micrograms/L concerning for developing diabetic ketoacidosis (DKA) and diffuse intravascular coagulation (DIC). A troponin of 0.059 ng/mL, B-type natriuretic peptide (BNP) 869 pg/mL, lactate of 6.2 mmol/L, creatinine of 1.84 mg/dL, aspartate aminotransferase (AST) of 78 IU/L, and alanine aminotransferase (ALT) of 69 IU/L were concerning for end-organ dysfunction. Thyroid-stimulating hormone (TSH) was normal at 2.67 mIU/L and free thyroxine was normal at 1.31 ng/dL.

With the presumptive diagnosis of pheochromocytoma crisis, the patient was given a 1 mg dose of phentolamine intravenously which rapidly normalized his blood pressure for about 15 minutes and was noted to be far more effective than the previously attempted nicardipine and nitroglycerin infusions at maintaining normotension. Based on this initial response, he was started on intravenous phentolamine drip at 4 mg per hour. A heparin infusion was also started for pulmonary emboli. The patient was transferred to the medical intensive care unit where his blood pressure became more labile with a sudden hypotensive period. Bedside ultrasound was used to determine there was free fluid in the abdomen, and a CT scan confirmed that he had suffered tumor capsule hemorrhage. He underwent emergent laparotomy for adrenalectomy with massive transfusion and survived to discharge after 14 days with confirmed histology of pheochromocytoma.

## Discussion

This patient presented in a pheochromocytoma crisis, which can be further separated into type A and type B. Type A crisis includes hemodynamic instability and evidence of multiple end-organ dysfunctions. Type A may progress to type B, which is differentiated by sustained hypotension and is associated with up to a 28% mortality rate [2,3]. 

While the clinical syndrome of hypertensive emergency with flash pulmonary edema was evident early in the case, the differential for what caused this state was broad. Since the patient was young and previously healthy, our suspicion for a secondary cause of hypertension was high and included pheochromocytoma, thyrotoxicosis, stimulant drug use, and even pancreatitis with concomitant pulmonary edema and acute respiratory distress syndrome given his initial presenting symptoms of epigastric abdominal pain, nausea, and vomiting. However, an older patient with more medical comorbidities with similar complaints may not have as robust of a tachycardic response, and be more likely to appear as having a primary cause of the hypertensive crisis. This could easily deceive an emergency physician into believing standard anti-hypertensive medications and cardiac preload augmentation will be sufficient to stabilize the patient, and they may also underestimate the degree of coexisting metabolic and coagulopathic derangements the patient may carry. 

In a hypertensive crisis with a pheochromocytoma, intravenous phentolamine provides the optimal blockade of catecholamine-induced vasoconstriction as a non-selective alpha-receptor blocker which may be given as an initial test dose of 1 mg followed by repeat 5 mg boluses or continuous infusion at 0.5-1 mg/minute [4]. In this case, we noted how long the initial 1 mg dose was effective for this particular patient and used this to determine the hourly infusion rate. If phentolamine fails to adequately control blood pressure, nicardipine may be added if not already started earlier in the patient’s undifferentiated course. Beta blockade should only be considered after adequate alpha blockade has been initiated to avoid unopposed alpha stimulation.

Endotracheal intubation is a noxious stimulant that the pheochromocytoma patient may respond unpredictably to given catecholamine levels that are already labile. Therefore, propofol and rocuronium are recommended for sedation and paralysis respectively in order to avoid further indirect sympathetic stimulation and histamine release [5].

Along with hypertensive emergency, this case demonstrates that management of pheochromocytoma can be complicated by metabolic derangements such as DKA, coagulopathic states such as DIC, and thromboembolic events. Pulmonary embolism in pheochromocytoma has been documented in several case reports along with other manifestations such as cerebral venous thrombosis and inferior vena cava thrombosis [6,7]. While a prothrombotic state is well known in the setting of malignancy, the exact pathophysiology of increased prevalence of thrombotic events in histologically benign pheochromocytoma is unknown. The use of heparin or other anticoagulants in the setting of a large adrenal tumor may be indicated, but used with caution due to the risk of adrenal capsule rupture. The size and speed of growth of an adrenal tumor and whether or not there is existing intratumoral hemorrhage should be considered by the physician to evaluate the risk of this possibly unavoidable complication, especially if the pheochromocytoma patient has a prolonged stay in the emergency department or transport time. Typically, adrenal tumors over 4 cm in size would be candidates for surgical resection as an outpatient, and it may be reasonable based on several case reports of spontaneous adrenal hemorrhage that tumors of this size, particularly pheochromocytomas, should be considered at increased risk of rapid expansion and capsular rupture [8]. This complication is especially catastrophic as emergent laparotomy in these cases may carry a mortality of 29% and a 100% mortality if the condition is not recognized and managed in a timely manner [9]. 

## Conclusions

Patients with undiagnosed pheochromocytoma may present in extremis to an emergency department with pheochromocytoma crisis and coexisting metabolic, thromboembolic, or surgically emergent events. Rapid diagnosis may be difficult, but can significantly alter the outcome in these critically ill patients. For this reason, emergency physicians should maintain a high index of suspicion for this elusive diagnosis and its potentially catastrophic complications. Alpha blockade with phentolamine is a mainstay of treatment that is infrequently used for other causes of hypertensive emergencies that emergency physicians should be familiar with. These patients ultimately require coordination with complex critical care and optimization for definitive surgical management, which begins in the emergency department.
